# Feasibility of home-based exercise training during adjuvant treatment for metastatic castrate-resistant prostate cancer patients treated with an androgen receptor pathway inhibitor (EXACT)

**DOI:** 10.1007/s00520-023-07894-1

**Published:** 2023-07-04

**Authors:** Malcolm Brown, Marie H. Murphy, Helen McAneney, Ken McBride, Ffiona Crawford, Aidan Cole, Joe M. O’Sullivan, Suneil Jain, Gillian Prue

**Affiliations:** 1 School of Nursing and Midwifery, Medical Biology Centre, Queen’s University Belfast, 97 Lisburn Road, Belfast, BT9 7BL UK; 2 Centre for Exercise Medicine, Physical Activity and Health, School of Sport, Ulster University, Belfast, UK; 3 School of Education and Sport, The University of Edinburgh, Scotland, UK; 4 Northern Ireland Public Health Research Network, School of Medicine, Ulster University, Belfast, UK; 5 School of Nursing, Midwifery and Health Systems, University College Dublin, Dublin, Ireland; 6 Northern Ireland Cancer Research Consumer Forum, Belfast, UK; 7 The Northern Ireland Cancer Centre, Belfast Health and Social Care Trust, Belfast, UK; 8 The Patrick G. Johnston Centre for Cancer Research, Queen’s University Belfast, Belfast, UK

**Keywords:** Exercise, Feasibility, Metastatic prostate cancer, Functional fitness, Health-related quality of life

## Abstract

**Background:**

Exercise is an effective adjuvant therapy that can alleviate treatment-related toxicities for men with prostate cancer (PC). However, the feasibility of delivering exercise training to men with advanced disease and the wider impact on clinical outcomes remain unknown. The purpose of the EXACT trial was to determine the feasibility and effects of home-based exercise training in men with metastatic castrate-resistant prostate cancer (mCRPC).

**Methods:**

Patients with mCRPC receiving ADT + an androgen receptor pathway inhibitor (ARPI) were prescribed 12 weeks of home-based, remotely monitored, moderate intensity, aerobic and resistance exercise. Feasibility was assessed using recruitment, retention and adherence rates. Safety and adverse events were monitored throughout, with functional and patient-reported outcomes captured at baseline, post-intervention and at 3-month follow-up.

**Results:**

From the 117 screened, 49 were deemed eligible and approached, with 30 patients providing informed consent (61% recruitment rate). Of those who consented, 28 patients completed baseline assessments, with 24 patients completing the intervention and 22 completing follow-up (retention rates: 86% and 79% respectively). Task completion was excellent throughout, with no intervention-related adverse events recorded. Self-reported adherence to the overall intervention was 82%. Exercise training decreased mean body mass (−1.5%), improved functional fitness (> 10%) and improved several patient-reported outcomes including clinically meaningful changes in fatigue (*p* = 0.042), FACT-G (*p* = 0.054) and FACT-P (*p* = 0.083), all with moderate effect sizes.

**Conclusion:**

Home-based exercise training, with weekly remote monitoring, was feasible and safe for men with mCRPC being treated with an ARPI. Given that treatment-related toxicities accumulate throughout the course of treatment, and as a result, negatively impact functional fitness and health-related quality of life (HRQoL), it was positive that exercise training improved or prevented a decline in these clinically important variables and could better equip patients for future treatment. Collectively, these preliminary feasibility findings support the need for a definitive, larger RCT, which downstream may lead to the inclusion of home-based exercise training as part of adjuvant care for mCRPC.

**Supplementary Information:**

The online version contains supplementary material available at 10.1007/s00520-023-07894-1.

## Introduction

Prostate cancer (PC) is the most commonly diagnosed male malignancy in Europe, with approximately 340,000 new cases annually [1]. With the adoption of prostate-specific antigen (PSA) screening programmes and improved awareness, most cases present with localised disease. However, a smaller proportion (~20%) are diagnosed with advanced disease characterised by metastatic progression to secondary sites, or they eventually enter this state having failed primary treatment. Androgen deprivation therapy (ADT) is the mainstay of advanced PC treatment and despite initial sensitivity, almost all patients progress to metastatic castration-resistant (mCRPC) disease [2]. Patients with mCRPC can now avail of a number of approved therapies, with individual treatment pathways involving cytotoxic chemotherapy, androgen receptor pathway inhibitors (ARPIs) and radionuclide therapies [3]. Despite these promising therapies and improving 5-year survival rates, mCRPC remains incurable [4].

Second-generation anti-androgens (e.g., abiraterone acetate or enzalutamide), known as ARPIs, combined with luteinising hormone-releasing hormone agonist (LHRHa) therapy are standard of care for mCRPC and confer significant survival benefits [5]. However, a substantial number of men experience an array of treatment-related toxicities including fatigue, increased adiposity, impaired bone health, decreased muscle mass/aerobic fitness and depression [6–10]. Such debilitating side effects ultimately lead to a poorer quality of life. With the increasing incidence of PC, alongside better overall survival, more men are now living with mCRPC and its treatment-related toxicities. Thus, managing this patient population poses a significant challenge for clinicians, with a strong emphasis placed on delaying progression, counteracting side effects and improving the overall quality of survivorship [11]. Exercise training can induce a host of beneficial physiological and psychological responses and has shown promise in alleviating this treatment burden [12–15].

Initial evidence advocating exercise training for patients with PC was collected from men receiving ADT for localised disease. Trials demonstrated exercise was safe and effective in improving muscular strength, cardiorespiratory fitness, physical function, lean body mass, quality of life and fatigue while actively receiving treatment [16–19]. However, clinical exercise trials in advanced PC remain limited. Preliminary evidence shows supervised exercise training for patients with metastatic disease shares similar benefits in terms of improved physical function, lean body mass and muscular strength compared to usual care [20, 21]. Importantly, exercise training in this advanced group is safe and well tolerated, with high compliance and retention rates [20]. While successful, exercise delivered onsite and supervised by exercise specialists is a resource-intensive model that is potentially unsustainable in the UK National Health Service (NHS) that is already facing widespread economic challenges [22]. Coupled with this issue, supervised exercise requires a greater time commitment, tends to be less flexible and poses obvious barriers that restrict accessibility, including proximity and cost of attendance [23]. 

Home-based, remotely managed, exercise training is emerging as an effective method of delivery during cancer treatment, and it has proved an acceptable alternative with high retention and improved metabolic and inflammatory profiles in localised PC receiving ADT [24, 25]. Recent publications have addressed the feasibility of home-based exercise training in mCRPC, demonstrating no safety concerns and good adherence and tolerance [26, 27]. However, neither included a contingent of UK patients nor followed up beyond their respective programmes, so potential chronic adaptations remain unknown. Further, it is currently unclear how this intervention might be implemented within the NHS where geographic and system-based differences exist (e.g., weather conditions, attitudes towards exercise, access to exercise specialists and private medical insurance versus government-sponsored universal healthcare). Therefore, the feasibility and extent to which these patients are willing to engage in exercise training remains to be determined. Furthermore, the need to evaluate this programme is essential given that this local intervention acts as a parallel alternative for the Movember INTERVAL-GAP4 global RCT [28]. This global trial involves 2 years of supervised, high-intensity interval training to determine the impact of exercise on overall survival. We anticipated that some men with mCRPC may be ineligible or unable to tolerate the exercise component (i.e., high-intensity training) of this larger trial, so we aimed to provide greater opportunity to access the benefits of regular, structured exercise, albeit at moderate intensity. Thus, our primary objective was to establish the feasibility of delivering a 12-week home-based, moderate-intensity exercise programme to men with mCRPC who are unable or ineligible for high-intensity exercise. The scientific hypothesis for EXACT is that home-based exercise training is feasible and can assist in managing treatment-related toxicities in mCRPC.

## Methods

### Design

**Fig. 1:**
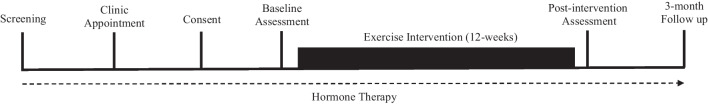
Trial timeline from screening to trial completion

This single-site, single-arm feasibility study (NCT03658486) examined the effects of 12 weeks of home-based/remotely supervised exercise training in men actively receiving ADT + an ARPI for mCRPC. The target sample size for this feasibility trial was 30 men, in accordance with recommendations [29]. Patients were identified by their clinical oncologist while attending their routine clinic appointment at The Northern Ireland Cancer Centre (Belfast City Hospital, Belfast Health and Social Care Trust) between January 2019 and February 2022. Following the confirmation of eligibility, patients attended three testing sessions at baseline, post-intervention and 3-month follow-up (Fig. 1). Initially planned as onsite, face-to-face visits, the COVID-19 pandemic and associated restrictions necessitated a protocol modification to enable remote assessments under strict mitigation measures. The conditions for onsite and remote assessments were identical to ensure reproducibility, with the only difference being the change in location. The remainder of the methods will be reported as onsite, but we will acknowledge COVID-19-specific adjustments as and when they arise. This manuscript is reported in accordance with the Consolidated Standards of Reporting Trials (CONSORT) guidelines [29].

### Participants

Men with mCRPC received medical clearance to participate from their clinical oncologist. Inclusion criteria specified that all men had testosterone levels < 50 ng/dL, were currently receiving ADT, were prescribed an ARPI (abiraterone acetate or enzalutamide), had an ECOG performance status of 0–1 and were at least 4 weeks removed from any surgery and fully recovered. Exclusion criteria included: patients currently exceeding exercise recommendations for cancer [30]; brain metastases; current, active secondary malignancy; congestive heart failure or recent cardiovascular event; unstable angina; uncontrolled metabolic disease; and pain with exertion. All patients provided informed consent to participate after reviewing the patient information sheet and having had the opportunity to ask any questions. PC diagnosis and treatment history were extracted from medical records. Ethical approval was obtained from the Office for Research Ethics Committees Northern Ireland (REC B, Reference: 18/NI/0108). Research governance permissions were granted by the Belfast Health and Social Care Trust (Reference: 18049GP-SS). All trial procedures were performed in accordance with the Declaration of Helsinki.

### Exercise intervention

This 12-week, home-based intervention has been described previously [31] and consisted of progressive, moderate-intensity walking and resistance exercise, 2–5 times per week (supplementary materials table 1). This intervention was designed in consultation with the ACSM exercise guidelines for oncology patients [30], with the aim of achieving these by week 9. Participants had the flexibility to complete walking and resistance exercises consecutively or separately based on readiness (e.g. symptom burden on any given day) or preference, and modifications were prescribed if necessary (e.g. metastatic bone lesions). Resistance training was performed using body mass and dumbbells (or weighted household items depending on dumbbell accessibility). Participants were provided with a guided warm-up and stretching exercises prior to completing exercise training. During the initial assessment, all exercises were demonstrated, and participants were provided with a pedometer (Digi-Walker, Yamax) to determine step count during exercise, an exercise booklet with further instructions and an exercise diary to log each training session. Participants reported their rate of perceived exertion (RPE) during each session to ensure they maintained an appropriate exercise intensity, using the 6–20-point scale, with each aiming for 12–14 during exercise [32]. Participants were encouraged to work beyond prescribed exercise if treatment-related side effects permitted, but equally, they could reduce and catch up on missed exercise when toxicities have subsided (i.e. autoregulation [33]). Weekly telephone contact between the exercise professional and participants acted as behavioural support and enabled remote monitoring, query resolution and guidance on exercise selection and progression for the duration of the exercise programme (i.e. 12 weeks). Exercise adherence was extracted from each exercise diary upon completion. Interruptions to the programme were documented if patients missed three consecutive sessions. Following the intervention, participants entered a self-managed maintenance phase (12 weeks), during which they were instructed to maintain their exercise regime of brisk walking and home-based resistance exercise in accordance with exercise recommendations.

### Feasibility outcomes

Feasibility was determined by the number of patients recruited during the 3-year recruitment window, as well as retention and adherence rates and response rates to patient-reported outcomes. All variables are expressed as percentages, with adherence reflecting the number of walking minutes and resistance repetitions completed versus prescribed. Intervention fidelity (i.e., the prescribed dose and any deviations/escalations from the protocol) was determined and the rate of adverse events in response to exercise or treatment, from the point of informed consent. Intervention-related adverse events were graded and coded according to the Common Terminology Criteria for Adverse Events (CTCAE, version 4.0).

### Body composition

Height and weight were determined using a free-standing stadiometer and calibrated laboratory scales, respectively. Body mass index was derived from these measurements (kg/m^2^). Hip and waist circumference was measured in centimetres using a tape measure. Anthropometric assessments were captured by the same investigator throughout the trial.

### Functional outcomes

To provide an indication of functional fitness, patients completed a timed 6-min walk test on a flat, indoor, 20-metre walkway. The 6-min walk test is a valid and reliable assessment in clinical populations and a surrogate measure of aerobic fitness [34]. Patients were instructed to walk briskly for the duration of the test. Heart rate response was monitored throughout, with perceived exertion rated at the end of the test. To provide an indication of lower extremity strength, a timed sit-to-stand test was used. This 30-second sit-to-stand test is a valid and reliable measure of lower extremity strength [35]. Patients were instructed to rise from a seated position to standing upright and return to seating, without assistance, as many times as possible within 30 seconds.

### Patient-reported outcomes

The severity and impact of pain on daily living, over a recall period of 24 h, were measured using the Brief Pain Inventory Short Form [36]. HRQoL was measured using the EuroQOL 5-dimension 5-levels (EQ-5D-5L) and Functional Assessment of Cancer Therapy-Prostate (FACT-P) questionnaires. The EQ-5D-5L questionnaire assesses HRQoL across five domains (mobility, self-care, usual activities, pain/discomfort and anxiety/depression) and provides a visual analogue scale for patients to self-assess their own health status [37]. The FACT-P is a 39-item HRQoL questionnaire assessing five domains (physical well-being, social/family well-being, emotional well-being, functional well-being and additional concerns) in the previous 7 days, with higher scores indicating improved quality of life [38]. Fatigue was assessed using the Functional Assessment of Chronic Illness Therapy-Fatigue (FACIT-fatigue) with higher scores indicating less fatigue [39]. Patients recalled and self-reported their physical activity levels (frequency and duration of vigorous intensity, moderate intensity, walking and sitting) during the previous 7 days using the International Physical Activity Questionnaire (IPAQ)-Short Form [40].

### Data analysis

For feasibility, the number of patients screened, those accrued and those who were not willing to participate with reasons was recorded. Attendance (for outcome assessments), compliance and retention rates for the intervention were analysed using descriptive statistics and reported as a percentage of their expected overall involvement. The acceptability of functional capacity and patient-reported outcomes was reported using completion rates. All measures were scored according to standard practice and analysed using paired sample *t*-tests to detect any changes. Effect size (Cohen’s *d*) was calculated for each with small, medium and large effects defined as 0.2, 0.5 and 0.8, respectively. Outcome data is presented as mean and standard deviation (95% confidence intervals), with clinically meaningful differences (according to normative data) noted. Data analysis was performed using SPSS version 29. Statistical significance was set at *p* < 0.05.

## Results

### Eligibility and recruitment rate

**Fig. 2:**
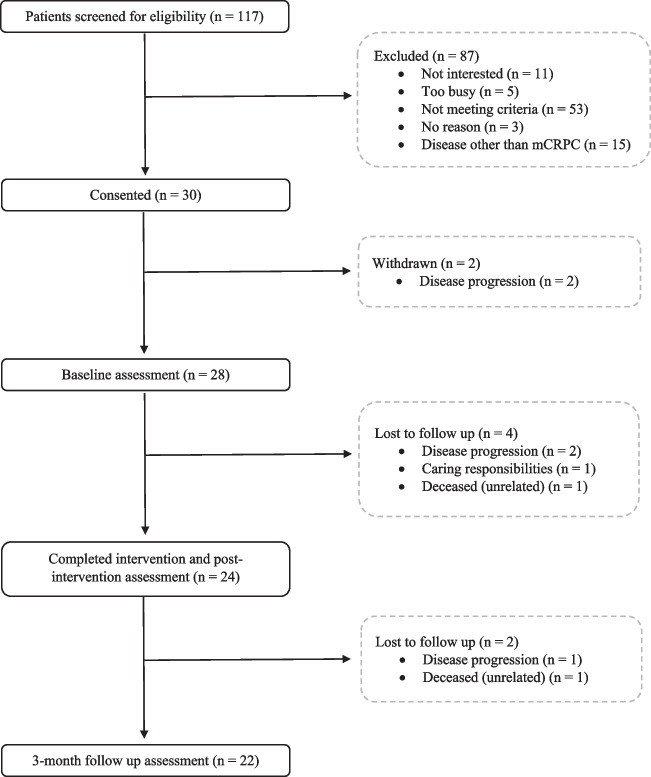
CONSORT diagram of recruitment and loss to follow up during the trial

In our 3-year recruitment window, one hundred and seventeen patients with advanced prostate cancer were assessed for eligibility by their treating clinician (Fig. 2). Recruitment and loss to follow-up are detailed in Fig. 2. Forty-five percent (*n* = 53) were excluded due to not meeting the eligibility criteria or alternatively at the discretion of the oncologist due to contraindications (e.g., comorbidities, frailty, disease progression and potential for non-compliance), while 13% (*n* = 15) were excluded for having a disease state other than mCRPC (e.g., hormone-sensitive). Among the men that declined (*n* = 19), the most common reasons were uninterested (58%) or too busy and lack of time (26%). Of those eligible and approached (*n* = 49), thirty patients were consented with 93% (*n* = 28) completing baseline testing and enrolling in exercise training. Thus, the recruitment rate (the proportion enrolled versus eligible) for this trial was 61%. It should also be noted that recruitment to this trial was severely impacted by several waves of the COVID-19 pandemic and associated restrictions within the UK, including a suspension to recruitment for all clinical trials (23 March 2020–15 September 2020). Interestingly, the initial 12 patients consented for EXACT were deemed ineligible by their treating clinician (*n* = 10) or excluded during screening (*n* = 2) for the INTERVAL-GAP4 trial. Due to the unforeseen suspension in recruitment and delays in reopening as a recruitment site for this global trial, all remaining patients consented were immediately referred to EXACT. No demographic differences (i.e., age, sex or race) existed between those that agreed to participate and those that declined the invite to participate.

### Participant characteristics

The mean age of participants was 71 years (range: 55–82 years). Mean BMI was 29.4 kg/m^2^ (range: 23.4–39.3 kg/m^2^) with 86% overweight (> 25 kg/m^2^) at baseline. Eighty-two percent of participants were married, 75% completed formal education and 71% were retired at trial entry. Most participants had received at least one prior treatment modality (86% radiotherapy; 32% chemotherapy), half had at least one comorbidity (57%) and 71% reported they had never smoked. All participants identified as white (British, Irish or European) (Table 1).

**Table 1:** Patient characteristics at baseline (*n* = 28)

Variables	Mean (standard deviation)
Age (years)	71 (6)
Height (cm)	175 (5)
PSA level at enrolment (ng/mL)	9.48 (15.6)
Prior treatment	
Radiotherapy	24
Chemotherapy	9
Surgery	5
Gleason grade	
2 − 6	0
3 + 4	1
4 + 3	9
8 − 10	18
Metastasis at enrolment	
Lymph nodes	15
Bone	21
Lung	1
Systemic therapies	
LHRHa	
Goserelin (Zoladex)	12
Triptorelin (Decapeptyl)	16
ARPI	
Abiraterone acetate	15
Enzalutamide	13
Comorbidities	
Diabetes type II	3
Cardiovascular disease	3
Hypertension	4
Osteoarthritis	3
Respiratory disease	1
Stomach disease	1
Ankylosing spondylitis	1
Race	
White	28
Education	
High school	6
Further education	5
Higher education	7
Other	3
None	7
Smoking status	
Current	2
Former	6
Never	20
Employment status	
Full-time	1
Part-time	5
Retired	20
Long-term sick leave	1
Casual/seasonal	1
Marital status	
Single	1
Married	23
Living with partner	1
Separated	1
Divorced	1
Widowed	1

*cm* centimetres, *ng/mL* nanograms per millilitre, *LHRHa* luteinising hormone-releasing hormone agonist, *ARPI* androgen receptor pathway inhibitor

### Retention and adherence rates

Eighty-six percent (*n* = 24) of patients that completed baseline outcomes went on to complete the 12 weeks of exercise training and post-intervention outcomes. Positively, 79% (*n* = 22) attended at a 3-month follow-up. All (100%) of the participants attempted and completed anthropometric and physical testing as well as patient-reported outcomes at baseline and post-intervention (no missing data). Task completion for the 6-min walking test at 3 months decreased slightly (96%) due to a single patient suffering ankle pain from an industrial-related accident. Aside from this, task completion for the remaining outcomes at 3 months (body composition and patient-reported outcomes) remained high (100%). The total number of outcome assessments through the duration of this trial was 74 (59% completed at treatment site, while 41% were completed remotely under identical conditions). Self-reported adherence to the overall intervention was 81.9%. Adherence to the aerobic component (i.e., brisk walking mins per week versus prescription) was 80.8%, while resistance training was 83% (based on the minimum threshold of repetitions per exercise).

### Intervention fidelity

Exercise training was interrupted on nine occasions during the intervention (Table 2). The most common reason for interruption was disease-/treatment-related toxicities including fatigue and bone pain (33%), followed by viral infection (22%). Of the 288 total training weeks, the intended weekly dose of aerobic exercise was modified on 98 occasions (34%) but encouragingly escalated on 162 weeks (56%) allowing patients to recover from the missed exercise. In terms of the aerobic component, patients were prescribed a cumulative dose of 1190 mins and completed 1613 ± 1128 mins (available in supplementary materials). Fifty-four percent (*n* = 13) of patients exceeded the planned dose of aerobic exercise during the 12-week intervention, while six (25%) were unable to achieve at least 1000 min due to a combination of work/lack of time (*n* = 3) and disease-/treatment-related factors (*n* = 3). Overall, 96% of patients attempted to complete aerobic exercise during the intervention. For resistance training, patients were prescribed a minimum cumulative dose of 2304 repetitions and completed 4092 ± 4030 repetitions (available in supplementary materials). Seventy-one percent (*n* = 17) attempted all prescribed resistance exercises, with all patients completing at least half of those prescribed. Comparable with aerobic exercise, 67% (*n* = 16) completed more repetitions than prescribed during the intervention. The weekly volume of both aerobic and resistance training versus prescribed is available in the supplementary materials (Figs. 1, 2, 3 and 4).

**Table 2:** Tolerability to exercise training in men with mCRPC

Variable	*N*	Pct. (%)
Permanent discontinuation (from consent)	6	20
Health-related		
Disease progression	4	67
Death	1	17
Personal		
Caring responsibilities	1	17
Exercise interruption	9	-
Health-related		
Treatment-related	3	33
Infection	2	22
Unwell	1	11
Fracture	1	11
Non-health related	2	22
Mean number of AET sessions completed (of 55)	51	93
Number who completed > 70% AET sessions as or more than prescribed	20	77
Number of AET dose escalations (weeks)	162	56
Number of AET dose reductions (weeks)	98	34
Health-related		
Fatigue	22	22
Bone pain	30	29
Nausea	5	5
Non health-related		
Time constraints / other commitments	12	12
Vacation	12	12
Weather	7	7
Other/no reason	10	10
Mean number of RET sessions completed (of 24 minimum)	42	175
Number who completed > 70% RET sessions as or more than prescribed	19	79

*N* number, *Pct* percentage, *AET* aerobic exercise training, *RET* resistance exercise training

### Adverse events

No safety concerns related to the exercise intervention were identified during the trial. One incident was reported outside of the exercise training, whereby a participant fractured his metatarsal during his daily routine (gardening). This occurred during week 2; however, he was able to resume exercise with a modified exercise programme at the start of week 9. Seventeen acute or ongoing disease-/treatment-related AEs were reported, resulting in missed exercise training. The most commonly cited treatment-related side effects were fatigue, bone pain and infection. Exercise training was permitted with fatigue, at a reduced level, but paused with more severe side effects until they subsided or completely resolved.

### Anthropometric outcomes

Exercise training decreased body mass post-intervention (*p* = 0.044; 95% CI = 0.04–2.51; Cohen’s *d* = 0.44). A mean decrease of 1.3 kg (−1.5%) was recorded post-intervention. No changes were detected at the 3-month follow-up (*p* = 0.841; 95% CI = 1.18–1.43; Cohen’s *d* = 0.04). Similarly, BMI decreased post-intervention (*p* = 0.045; 95% CI = 0.01–0.80; Cohen’s *d* = 0.43) but returned to baseline levels at 3 months. Waist and hip circumferences remained unchanged from baseline (Table 3).

**Table 3:** Changes in physical function, body composition and patient-reported outcomes before and following 12 weeks of exercise training

	Baseline	Post-intervention	SMD [95% CI]	3-month follow-up	SMD [95% CI]
Mass (kg)	90 (13)	89 (12)*	−1.0 [0.04-2.51]	90 (14)	0 [−1.18–1.43]
BMI (kg/m^2^)	29.4 (4)	29 (3.9)*	−0.4 [0.01–0.80]	29.4 (4.5)	0 [−0.38–0.44]
Hip (cm)	109 (5)	108 (5)	−1.0 [−0.55–2.63]	108 (5)	−1.0 [−0.53–2.40]
Waist (cm)	111 (11)	110 (12)	−1.0 [−0.63–3.17]	110 (13)	−1.0 [−0.66–2.79]
6-min walk (m)	451 (64)	511 (89)*	60 [34.1–85.1]	500 (74)*	49 [−63.4 to −30.9]
Timed sit-to-stand (reps)	12 (3)	15 (3)*	3 [2.17–4.08]	15 (2)*	3 [−3.77 to −1.22]
FACT-G	84 (20)	88 (19)	4 [−9.01–2.76]	91 (13)	7 [−14.1–0.14]
Physical	22 (7)	23 (6)	1 [−2.95–0.95]	23 (4)	1 [−3.87–0.32]
Social	24 (4)	24 (4)	0 [−2.07–0.98]	25 (3)	1 [−2.93–0.02]
Emotional	19 (6)	19 (6)	0 [−2.06–2.06]	23 (5)	1 [−4.05–1.32]
Functional	20 (6)	22 (5)	2 [−4.02–0.85]	20 (5)	3 [−5.33–0.52]
FACT-P	118 (29)	121 (27)	3 [−11.4–5.58]	126 (20)	8 [−17.9–1.17]
FACIT-fatigue	35.3 (13.3)	38.3 (12.5)	3 [−5.99–0.16]	39.4 (10.2)*	4.1 [−7.84 to −0.15]
BPI					
Severity	1.7 (2.6)	1.9 (2.3)	0.2 [−0.67–1.07]	1.7 (2.1)	0 [−1.09–1.18]
Interference	1.8 (2.8)	1.8 (2.3)	0 [−0.73–0.75]	1.5 (2.4)	−0.3 [−1.48–0.94]
EQ-5D-5L					
Mobility	2 (1)	2 (1)	0 [−0.39–0.39]	2 (1)	0 [−0.48–0.39]
Self-care	1 (1)	1 (1)	0 [−0.26–0.43]	1 (1)	0 [−0.24–0.60]
Usual activities	2 (1)	2 (1)	0 [−0.40–0.32]	2 (1)	0 [−0.38–0.38]
Pain/discomfort	2 (1)	2 (1)	0 [−0.57–0.57]	2 (1)	0 [−0.55–0.64]
Anxiety/depression	2 (1)	2 (1)	0 [−0.46–0.21]	1 (1)^†^	−1 [−0.01–0.92]
VAS	72.5 (21.2)	75 (17.1)	2.5 [−10.3–5.37]	74.7 (19.2)	2.2 [−10.3–5.54]
Physical activity					
Vigorous (d·wk^−1^)	0.3 (0.8)	1.8 (2.4)*	1.5 [−2.52–0.38]	1.1 (2.1)	0.8 [−1.81–0.17]
ime (min·day^−1^)	10.6 (33.8)	49.1 (95.5)	38.5 [−82.3–6.31]	30.9 (56.2)	20.3 [−44.6–6.02]
Moderate (d·wk^−1^)	1.9 (2.7)	3.8 (2.8)*	1.9 [−3.29–0.45]	3.2 (2.6)	1.3 [−2.76–0.39]
Time (min·day^−1^)	31.5 (63.5)	71.6 (93.3)	40.1 [−97.5–12.3]	81.1 (90.8)*	49.6 [−91.5 to −8.49]
Walking (d·wk^−1^)	4.8 (2.4)	5.5 (2)	0.7 [−1.6–0.3]	4.5 (2.8)	−0.3 [−0.95–1.41]
Time (min·day^−1^)	59.2 (64.4)	60 (68.9)	0.8 [−31.2–24.3]	52 (49)	−7.2 [−20.2–35.7]
Sitting (h·day^−1^)	7.3 (3)	6.1 (3)*	−1.2 [0.28–2.36]	6.2 (2.6)	−1.1 [−0.06–2.89]

Data expressed as mean (SD). Abbreviations: *SMD* standard mean difference [from baseline], *CI* confidence interval, *BMI* body mass index, *BPI* brief pain inventory, *d·wk*^***−****1*^ days per week, *EQ-5D-5L* EuroQol 5-dimensions 5-levels, *VAS* visual analogue scale, *FACT-G* functional assessment of cancer therapy-general, *FACT-P* functional assessment of cancer therapy-prostate, *IPAQ* international physical activity questionnaire, post min·day^**−**1^, mins per day

*represents a statistically significant change versus baseline (*p* < 0.05)

^†^represents a statistically significant change versus post-intervention (*p* < 0.05)

### Physical outcomes

Exercise training improved 6-min walking distance post-intervention (+13.3%) (*p* < 0.001; 95% CI = 34.1–85.1; Cohen’s *d* = 0.99) and at 3-month follow-up (+11.1%) (*p* < 0.001; 95% CI = −63.4 to −30.9; Cohen’s *d* = 1.32). Similar improvements in lower extremity muscular strength were detected, with increased repetitions for the timed sit-to-stand test at post-intervention (+25%) (*p* < 0.001; 95% CI = 2.17–4.08; Cohen’s *d* = 1.39) and 3-months (+25%) (*p* < 0.001; 95% CI = −3.77 to −1.22; Cohen’s *d* = 0.87) (Table 3).

### Patient-reported outcomes

Exercise training improved fatigue at the 3-month follow-up compared to baseline (*p* = 0.042; 95% CI = −7.84 to −0.15; Cohen’s *d* = 0.46). Following the intervention, the anxiety/depression dimension of EuroQoL decreased at 3-month follow-up, compared to post-intervention (*p* = 0.015; 95% CI = 0.12–0.97; Cohen’s *d* = 0.57). No changes were detected between baseline and 3 months for anxiety/depression (*p* = 0.057) or the remaining four dimensions. Self-reported physical activity levels (number of days) increased for both vigorous (*p* = 0.01; 95% CI = −2.52–0.38; Cohen’s *d* = 0.58) and moderate exercise (*p* = 0.012; 95% CI = −3.29–0.45; Cohen’s *d* = 0.56) at post-intervention while sitting hours decreased (*p* = 0.015; 95% CI = 0.28–2.36; Cohen’s *d* = 0.55). The duration of moderate-intensity minutes also increased at 3-month follow-up compared to baseline (*p* = 0.021; 95% CI = −91.5 to −8.49; Cohen’s *d* = 0.55). Finally, no changes were detected in pain severity, pain interference or overall scores for the FACT-G/FACT-P (Table 3).

## Discussion

We have shown that home-based, concurrent exercise training is safe and feasible for men with mCRPC. To our knowledge, this trial recruited, retained and completed the largest sample of advanced prostate cancer patients to a home-based exercise intervention to date, in the UK and globally. Exercise training has been shown for the first time to result in improved body mass, functional fitness and patient-reported outcomes, particularly fatigue, in a sample of UK patients with mCRPC. This trial served as a parallel alternative to the INTERVAL-GAP4 trial in Belfast, highlighting that if men were deemed ineligible, then they could still participate and complete a moderate intensity exercise programme. This is important from a patient care perspective, as they could avail of the benefits of being active (i.e., improved fitness, reduced fatigue and improved HRQoL), albeit at a moderate intensity and while at home.

In terms of feasibility, our recruitment rate from the eligible population was 61%. When we acknowledge that many men were excluded due to ineligibility/unsuitability and recognise the landscape of recruitment during several waves of COVID-19 lockdown and restrictions, as well as competing trials (i.e., INTERVAL-GAP4), this recruitment rate is encouraging. Moreover, it indicates demand and willingness in men with advanced prostate cancer to engage in a lifestyle-related intervention while receiving ongoing treatment. Compared to other exercise trials in PC, our recruitment rate was lower than some interventions [20, 24, 41], equal to others, including supervised [21, 42], but crucially greater than other interventions incorporating remote options [25–27]. It is important to consider the volume of patients screened or referred when determining recruitment rates, with greater numbers seemingly resulting in lower recruitment rates and highlighting a need for a streamlined referral pathway. Of those screened (*n* = 117), 49 patients (42%) were approached by their clinician with the opportunity to participate, suggesting strong clinical support for this intervention. Sixty-one percent (*n* = 30) of those approached provided informed consent and were enrolled, with 93% (*n* = 28) attending for baseline assessment. The retention rate for this intervention was strong, standing at 86% post-intervention and 79% at follow-up (from baseline). Compared to other home-based trials [25–27], our retention rate is greater and similar to those reported during supervised interventions [20, 21, 42], further reiterating a determination to engage while facing persistent toxicities. While our retention is on par, supervised interventions have greater adherence (89–93%), although this current trial has greater compliance than others offering solely home-based or home-based as an intervention arm [25, 27]. Compared to other home-based interventions [25–27], we also have the greatest task completion of physical and patient-reported outcomes at baseline, post-intervention and follow-up. Pragmatically, we scheduled outcome measures to coincide with routine clinical visits and conducted assessments within our co-located exercise facility, providing continuity in treatment and minimising the burden placed on patients, likely leading to the high attendance and completion rates observed. In agreement with similar trials in PC, no intervention-related adverse events were reported [20, 21, 27, 41, 42]. Collectively, our findings alongside other trials suggest that exercise training is safe and feasible and could be delivered as an adjuvant therapy to support patients with mCRPC. Within the UK National Health Service implementation of supervised exercise could prove problematic (e.g., economic and staffing [22]), hence the rationale for this cost-effective, home-based, remotely managed alternative to maintain safety, retention and compliance. This strategy does not place any further economic strain on the health service, is flexible and inclusive, and is perhaps more palatable to patients who are already burdened by ongoing treatment.

Tolerability of exercise training is equally important as feasibility and practicality for patients diagnosed with mCRPC. This trial demonstrates the fidelity of delivering home-based concurrent exercise training as an adjunct therapy to compliment conventional treatment pathways. Throughout the course of treatment with ADT, it is to be expected that patients will face periods of heightened and reduced toxicities, and as a result, the exercise stimulus can be modified accordingly, as reflected in the observed dose interruptions, reductions and escalations [23]. Conceptually referred to as ‘autoregulation’, this process permits dose modifications based on individual readiness and was pivotal in delivering exercise for patients where the disease burden is complex (i.e., mCRPC) [43]. This flexibility can improve enjoyment and encourage longer-term maintenance, provide insightful evidence of the challenges in this population and may have contributed to the strong feasibility outcomes for this intervention [44]. It is reassuring that if exercise training was interrupted or reduced, most had the capacity to recover the exercise dose and/or escalate beyond the desired prescription. For aerobic exercise, over half of the patients (54%) exceeded the planned dose, while 67% completed more repetitions than minimally prescribed for resistance training. Cumulatively, patients completed 1613 ± 1128 min of aerobic exercise and 4092 ± 4030 repetitions of resistance exercise, which were 35% and 78% greater than minimally prescribed, respectively. The weekly mean volume for aerobic training was greater than prescribed for 92% of the intervention (11/12 weeks), while the mean volume per week for resistance exercise was greater throughout (available in supplementary materials).

Several studies have reported marked changes in body composition in men receiving ADT for prostate cancer [45, 46]. Testosterone suppression is associated with significant weight gain, with patients rapidly losing lean mass and gaining fat mass, particularly in the initial years of treatment [47–49]. BMI and in particular excess fat mass, is associated with poorer treatment responses and prostate cancer-specific mortality. Coupled with this ADT-mediated increase in fat mass, patients are also at a heightened risk of developing cardiometabolic complications [50, 51]. Positively, current exercise training assisted in reducing body mass, leading to an improved body mass index independent of ADT. This contrasts with a similar trial in mCRPC [27] but agrees with other exercise interventions showing improved body mass during ADT [52]. While the differentials of body composition were not measured as part of this trial, we cannot definitively determine the impact of exercise training. Instead, we can only speculate in light of findings from a recent systematic review of 116 articles and over 4000 patients. It was shown that concurrent exercise training (aerobic and resistance) effectively reduced body fat percentage and body fat mass, while resistance training effectively increased lean mass in overweight individuals [53]. As the mode of exercise training aligns with this trial and the mean body mass of this current population at baseline suggests they are overweight, it is conceivable that this might be the case. Interestingly, the impact of exercise training on body mass is amplified in patients exposed to ADT, if delivered as part of a comprehensive lifestyle intervention [54, 55], highlighting a possible avenue of future research in mCRPC. Nonetheless, the observed reduction in body mass with concurrent, home-based exercise training could positively alter the systemic inflammatory profile of patients (e.g., adipokines/myokines), beneficially impact QoL and prevent comorbidities including sarcopenia (in the event of increasing lean body mass).

Cardiorespiratory fitness is a sensitive mediator of treatment tolerance and symptom burden and is an independent predictor of survival [56–58]. Anti-cancer treatment alongside lifestyle factors and the natural aging process markedly impairs cardiorespiratory fitness, posing an important consideration for patients with advanced cancer. The 6-min walking test has proved a valid and reliable measure of functional capacity and is used as an indirect estimate of cardiorespiratory fitness in advanced cancer [34]. This trial has shown that exercise training improved functional fitness and muscular strength post-intervention, and encouragingly, these improvements persisted at a 3-month follow-up. Relative to normative values for 6-min walking, these men outperformed their breast cancer counterparts [59]. This is a consistent finding, with several reporting improved functional fitness post-intervention in PC, although almost exclusively with supervised exercise training [20, 21, 42, 52]. Another recent home-based intervention reported a moderate effect size for the 400-m walk but did not reach significance [27], possibly connected to a smaller sample size or perhaps slightly different testing conditions (e.g., 400-m walk to 6-min walking test). Regardless, this improvement in functional fitness prevented an inevitable decline in cardiorespiratory fitness and may have downstream effects on overall HRQoL. Moreover, patients diagnosed with mCRPC will inevitably progress, and thus, improved cardiorespiratory fitness should improve the tolerability of future treatment.

The systemic therapies utilised during the management of mCRPC can severely impact HRQoL. Encouragingly, exercise training improved fatigue at a 3-month follow-up, using the FACIT-fatigue scale. This clinically meaningful change (≥ 3 points) demonstrates that exercise training during ADT can offset this debilitating side effect and improve HRQoL. Given that cancer-related fatigue is the most commonly cited treatment-related side effect of PC that limits usual functioning [60], exercise training may prove an effective solution in attenuating the overall impact of this condition as well as the negative consequences associated with cancer-related fatigue (e.g., physical, emotional and cognitive distress). Taken alongside other findings as part of this trial, including a clinically meaningful change (3 points) in the functional well-being domain (FACT-P) and a decrease in the anxiety/depression dimension of EuroQoL, this appears to hold true. Likewise, clinically meaningful changes in both FACT-G (7 points; *p* = 0.054; Cohen’s *d* = 0.44) and FACT-P (6–10 points; *p* = 0.083; Cohen’s *d* = 0.39) total scores at 3-months further suggests an overall improvement in HRQoL. While such improvements are commonplace, our findings contrast with another trial in mCRPC that showed no changes (statistically or clinically meaningful) in either the FACIT-fatigue or FACT-P scales immediately post-intervention [27]. However, this parallel trial did not include follow-up outcome measures, suggesting such changes may present in the longer term for men with mCRPC. Providing patients suitably maintain a level of exercise training after the trial setting, these changes could assist in disease management and continue to improve/maintain HRQoL. Finally, it should also be noted that while some patient-reported outcomes remained unchanged, these too could be viewed in a positive light, given that HRQoL is expected to decline over the course of treatment, and thus exercise training may prevent this deterioration.

### Limitations

While the findings of this trial are positive, there are several limitations to consider. Firstly, this trial was designed as a single-arm intervention, with baseline outcomes as the comparator. Having no control group prevents comparisons with usual care. As a feasibility trial and thus a small sample, caution is advised regarding the interpretation of *p* values and inferences of exercise effectiveness - only a larger RCT will determine these conclusively [29]. This intervention was limited to 12 weeks in duration, which is common practice, although longer interventions are required (possibly from the start of ADT) to comprehensively evaluate feasibility outcomes in the future. Closely related, we placed no restrictions on enrolment criteria for these patients, aside from a diagnosis of mCRPC and actively receiving ADT + ARPI, meaning some remained stable while others progressed. Moreover, some patients had differing treatment durations (new/< 1 year/> 1 year) and different treatment options (e.g., abiraterone acetate or enzalutamide), which may have resulted in subtle differences based on disease trajectory. Stratifying findings in the future with a larger sample could clarify. All patients involved in this trial were white, married and educated. Future studies should attempt to evaluate feasibility and impact in a more diverse sample of different ethnicities and socioeconomic status, in accordance with NIHR INCLUDE guidance [61]. As this trial was individualised and home-based, patients self-selected the number of repetitions during resistance training based on their readiness (from a range provided), with little uniformity in the weight lifted. As such, the resistance dose is difficult to quantify and some of the true nuances of resistance training may have been overlooked [43]. Similarly, we did not directly assess how many patients continued to exercise or how much they completed during the self-managed maintenance phase. Instead, patients subjectively reported continued exercise and completed a 7-day recall at 3 months, introducing a level of reporting bias. Finally, as this trial was home-based, patients self-reported their level of activity with the assistance of a pedometer. Future studies should attempt to support self-reported activity with objective measures (e.g., accelerometer).

## Conclusion

In this feasibility study, we have demonstrated that home-based, remotely managed, exercise training was feasible in terms of recruitment, retention and adherence and safe in men with mCRPC being treated with ADT + ARPI. Exercise training positively impacted body mass, functional fitness and importantly patient-reported outcomes. Collectively, it appears that the benefits accrued from this intervention assisted in managing debilitating treatment-related toxicities associated with ADT and may better equip men for future treatment as a result. Thus, home-based exercise training could prove an effective and viable alternative to supervised exercise for men with mCRPC. Given that the number of men living with mCRPC is expected to rise as a result of new and emerging therapies extending survival, understanding their complex needs and the best supportive care strategies to improve outcomes is crucially important. Home-based exercise training is accessible and scalable and should form part of this care pathway in the future. A definitive, sufficiently powered RCT is now required to replicate these findings and establish the chronic effects of training, together with an investigation on the biological impact of exercise training on tumour cell growth kinetics [62].

## Supplementary information

ESM 1 (DOCX 36 kb)

## Data Availability

Data generated during this study is available from the corresponding author on reasonable request.
