# Supplementary material for: Feasibility of home-based exercise training during adjuvant treatment for metastatic castrate-resistant prostate cancer patients treated with an androgen receptor pathway inhibitor (EXACT)
Source: Support Care Cancer. 2023 Jul 4;31(7):442. doi: 10.1007/s00520-023-07894-1 (PMC10319656; doi:10.1007/s00520-023-07894-1)
Supplement: Supplementary file 1 — (DOCX 36 kb) [file 520_2023_7894_MOESM1_ESM.docx]

| Week | Aerobic Exercise (Brisk Walking) | Resistance Exercise | | | |
| --- | --- | --- | --- | --- | --- |
|  | **Mins per week (minimum)** | **Sets** | **Repetitions** | **Days per week** | **Exercises** |
| 1 | 20 | 1 | 8 - 15 | 2 - 3 | 1. Wall Press 2. Sit-to-stand |
| 2 | 30 | 2 | 8 - 15 | 2 - 3 | 1. Wall Press 2. Sit-to-stand |
| 3 | 50 | 3 | 8 - 15 | 2 - 3 | 1. Wall Press 2. Sit-to-stand |
| 4 | 70 | 3 sets (1 - 2)  1 set (3 - 4) | 8 - 15 | 2 - 3 | 1. Wall Press 2. Sit-to-stand 3. Lateral raise (arms) 4. Lateral raise (legs) |
| 5 | 70 | 3 sets (1 - 2)  2 sets (3 - 4) | 8 - 15 | 2 - 3 | 1. Wall Press 2. Sit-to-stand 3. Lateral raise (arms) 4. Lateral raise (legs) |
| 6 | 120 | 3 sets (1 - 2)  3 sets (3 - 4) | 8 - 15 | 2 - 3 | 1. Wall Press 2. Sit-to-stand 3. Lateral raise (arms) 4. Lateral raise (legs) |
| 7 | 120 | 3 sets (1 - 2)  3 sets (3 - 4)  1 set (5 - 6) | 8 - 15 | 2 - 3 | 1. Wall Press 2. Sit-to-stand 3. Lateral raise (arms) 4. Lateral raise (legs)  5. Bicep curl 6. Squat |
| 8 | 130 | 3 sets (1 - 2)  3 sets (3 - 4)  2 sets (5 - 6) | 8 - 15 | 2 - 3 | 1. Wall Press 2. Sit-to-stand 3. Lateral raise (arms) 4. Lateral raise (legs)  5. Bicep curl 6. Squat |
| 9 | 130 | 3 sets (1 - 2)  3 sets (3 - 4)  3 sets (5 - 6) | 8 - 15 | 2 - 3 | 1. Wall Press 2. Sit-to-stand 3. Lateral raise (arms) 4. Lateral raise (legs)  5. Bicep curl 6. Squat |
| 10 - 12 | **Maintenance phase**  AET - 30 mins on at least 5 days per week  RET - 3 - 4 sets; 6 exercises; 2 - 3 days per week | | | | |

**Table 1: Home-based exercise training programme for patients with mCRPC.** Patients completed both aerobic and resistance exercise at 12-14 on the 6-20-point rating of perceived exertion (RPE) scale. Abbreviations: AET, aerobic exercise training; RET, resistance exercise training.

**Fig. 1: Total cumulative dose of planned and completed aerobic exercise training (AET).**

**Fig. 2: Mean aerobic exercise training volume per week (prescribed versus completed).**

**Fig. 3: Total cumulative dose of planned and completed resistance exercise training (RET).** Planned based on the minimum threshold of 8 repetitions.

**Fig. 4: Resistance exercise training volume per week (planned versus completed).** Planned based on the minimum threshold of 8 repetitions.
